# Variation in the Assessment of Immune-Related Adverse Event Occurrence, Grade, and Timing in Patients Receiving Immune Checkpoint Inhibitors

**DOI:** 10.1001/jamanetworkopen.2019.11519

**Published:** 2019-09-18

**Authors:** David Hsiehchen, Mary K. Watters, Rong Lu, Yang Xie, David E. Gerber

**Affiliations:** 1 Department of Internal Medicine, University of Texas Southwestern Medical Center, Dallas; 2 Department of Population and Data Sciences, University of Texas Southwestern Medical Center, Dallas; 3 Harold C. Simmons Comprehensive Cancer Center, University of Texas Southwestern Medical Center, Dallas

## Abstract

**Importance:**

Toxic effects of conventional chemotherapy and molecularly targeted cancer therapies are generally well defined and occur at predictable points. By contrast, owing to their heterogeneous manifestations, unpredictable timing, and clinical overlap with other conditions, immune-related adverse events (irAE) may be more difficult to diagnose and characterize.

**Objective:**

To determine concordance of algorithm-driven medical record review by medical oncologists for the characterization of 8 irAE in patients treated with immune checkpoint inhibitors.

**Design, Setting, and Participants:**

Cross-sectional study of patients treated with immune checkpoint inhibitors at a National Cancer Institute–designated comprehensive cancer center from November 30, 2015, to March 7, 2018. A sample size of 52 patients provided 80% power to distinguish substantial agreement (κ = 0.85) from poor agreement (κ = 0.5) based on the Cohen κ.

**Main Outcomes and Measures:**

Interrater agreement of 2 observers in the occurrence and grade of irAE.

**Results:**

Of 52 patients (32 [61.5%] male; mean [SD] age, 69 [9] years) analyzed, 42 (80.8%) had non–small cell lung cancer and all received anti–programmed cell death 1 or anti–programmed cell death ligand 1 antibodies, with 3 patients (5.8%) receiving combinations with anti–cytotoxic T-lymphocyte antigen 4 antibodies. A median (interquartile range) of 82 (47-180) documents were reviewed per case. There was limited or poor interrater agreement on irAE occurrence (Cohen κ, 0.37-0.64), with the exception of hypothyroidism (κ = 0.8). Weighted κ similarly showed limited or poor agreement for irAE grade (κ = 0.31-0.75). Differences in assessed time of onset ranged from 5 to 188 days. As a control for data availability and access, observers had a high degree of agreement for the exact start date (98%) and end date (96%) of immunotherapy administration, suggesting that information interpretation rather than identification largely accounted for assessment differences. In multivariable analysis, therapy duration (adjusted odds ratio, 4.80; 95% CI, 1.34-17.17; *P* = .02) and Charlson Comorbidity Index (adjusted odds ratio, 4.09; 95% CI, 1.10-15.18; *P* = .03) were significantly associated with discordant irAE assessment.

**Conclusions and Relevance:**

These findings underscore critical challenges in assessing the occurrence, type, timing, and severity of irAE. Apart from hypothyroidism (a condition that has a discrete diagnostic laboratory test and few other likely etiologies during immunotherapy treatment), interobserver agreement was poor. Given the importance of accurate and timely assessment of toxic effects for clinical trials and real-world disease management, efforts to improve irAE diagnosis and characterization are needed.

## Key Points

**Question:**

How accurate are clinicians in diagnosing and characterizing immune-related adverse events from cancer immunotherapy?

**Findings:**

In a cross-sectional study using an algorithm-driven approach to characterize immune-related adverse events, poor concordance of interrater agreement was found in the occurrence, severity, and timing of 8 common immune-related adverse events. Discordance was associated with longer durations of therapy and higher comorbidity burden in patients.

**Meaning:**

These findings suggest that the diagnosis and characterization of immune-related adverse events are challenging and have direct relevance to immunotherapy clinical trials and the care of patients receiving immune checkpoint inhibitors.

## Teaser

This cross-sectional study assesses the concordance of algorithm-driven medical record review by medical oncologists for the characterization of 8 immune-related adverse events in patients with cancer treated with immune checkpoint inhibitors.

## Introduction

Recognizing and characterizing treatment-related adverse events (AE) represents a cornerstone of determining the value of oncology treatments for patients and health care professionals.^1^ A shortage of high-quality and reliable AE data, even among pivotal phase 3 trials, has prompted a call for more rigorous standards of AE reporting.^2,3,4^ The advent of immune checkpoint inhibitors likely adds considerable challenge to this effort. Toxic effects of conventional chemotherapy, in particular myelosuppression, can be documented and graded with standard laboratory values such as neutrophil or platelet counts. Conversely, immune-related adverse events (irAE) may involve almost every organ.^5^ These autoimmune toxic effects are unpredictable, possibly permanent, and occasionally fatal.^6^

Now that single-agent and combination immune checkpoint inhibitor regimens are in broad clinical use for multiple cancers, the timely and reliable diagnosis of irAE is critical for safe and effective disease management. Results from emerging, single-center experiences with immunotherapy consistently demonstrate higher irAE rates than those reported in prospective therapeutic clinical trials.^7,8,9,10^ Indeed, the reported incidence of pneumonitis is a magnitude greater in multiple real-world series than in clinical trials, suggesting that differences in patient populations do not fully account for such discrepancies. Rather, it seems likely that heterogeneous manifestations, unpredictable timing, and clinical overlap with other conditions contribute to challenges and differences in irAE characterization.^4,11^ However, to our knowledge, the accuracy of irAE diagnosis has not been evaluated. We therefore evaluated variation in the assessment of irAE occurrence, grading, and timing among clinicians.

## Methods

This study was approved by the University of Texas Southwestern institutional review board. Written informed consent was obtained from all participants. The study followed Strengthening the Reporting of Observational Studies in Epidemiology (STROBE) reporting guideline for cross-sectional studies. Using the EPIC electronic health record (Epic Systems Corp), 2 medical oncologists (D.H. and M.K.W.) experienced in the administration and assessment of immune checkpoint inhibitor–based therapy concurrently but independently reviewed data on consecutive patients who received immune checkpoint inhibitor therapy at the Harold C. Simmons Comprehensive Cancer Center and were enrolled in a prospective observational registry study from November 30, 2015, to March 7, 2018. To reduce variability in interpretation, manual abstraction of records occurred through a standard operating procedure developed to systematically extract the occurrence, grade, and timing of irAE. Medical record abstraction included all clinic notes, telephone encounters, radiology images and reports, laboratory results, medication lists, and hospitalization records. For instance, occurrence of pneumonitis was determined from content in clinic notes, hospitalization records, telephone encounters, and medication lists, as well as review of all interval chest radiology images and reports. Records were analyzed from 1 month prior to the initiation date of immunotherapy and up to 3 months after the last dose of immunotherapy to establish baseline measurements and capture delayed-onset irAE. Additionally, the last available medical oncology note was reviewed for the possibility of later-onset irAE and long-term sequelae.

Observers focused on 8 irAE (adrenal insufficiency, colitis, hepatitis, hyperthyroidism, hypophysitis, hypothyroidism, pneumonitis, and rash). Grade of irAE was based on the *Common Terminology Criteria for Adverse Events* version 5.0. Medical comorbidities were recorded and scored according to the Charlson Comorbidity Index.

### Statistical Analysis

Sample size for this analysis was determined as follows: assuming each patient had a 20% chance of developing irAE of interest,^7,8,9^ 52 patients provided 80% power to differentiate a substantial agreement (κ = 0.85) from a poor agreement (κ = 0.5) at a significance level of .05 using 1-tailed tests. Cohen κ and the weighted κ were used to measure interreviewer agreement on irAE occurrence and grade, respectively. Sample size estimation was performed by implementing the function N.cohen.kappa from the R statistical software package irr (R Project for Statistical Computing). Both Cohen κ and the weighted κ were calculated using the function ckap in the R package rel. The weighted κ was calculated with linear weighting. Odds ratios between case characteristics and irAE discordance were analyzed by univariable and multivariable logistic regression. The multivariable model included therapy duration, number of documents reviewed, Charlson Comorbidity Index, and history of autoimmune disease as predictor variables. Age, sex, and race were not significantly associated with discordance in univariable analyses and thus were excluded from the multivariable model. A Charlson Comorbidity Index value of 9 was used to stratify high and low comorbidity based on prior studies in patients with advanced cancer.^12,13^

## Results

Among the 52 patients included in the analysis, the mean (SD) age was 69 (9) years, 32 (61.5%) were male, and 42 (80.8%) had non–small cell lung cancer (Table 1). Treatment consisted of anti–programmed cell death 1 (PD-1) antibody (40 patients [76.9%]), anti–programmed cell death ligand 1 (PD-L1) antibody (9 patients [17.3%]), or anti–PD-1 plus anti–cytotoxic T-lymphocyte antigen 4 (CTLA-4) antibodies (3 patients [5.8%]). Median (interquartile range) duration of therapy (measured from date of first to last immune checkpoint inhibitor infusion) was 50 (15-304) days. Across cases, a median (interquartile range) of 82 (47-180) documents (defined as oncology clinic notes, telephone encounters, imaging studies, and hospitalization records, but not including laboratory results) were reviewed per patient.

**Table 1.** Patient Characteristics

Characteristic	No. (%)
Age, mean (SD), y	69 (9)
Sex	
Male	32 (61.5)
Female	20 (38.5)
Race	
White	46 (88.5)
Other	6 (11.5)
Cancer type	
Non–small cell lung cancer	42 (80.8)
Renal cell carcinoma	5 (9.6)
Melanoma	1 (1.9)
Other	4 (7.7)
Immune therapy	
Anti–PD-1 antibody	40 (76.9)
Anti–PD-L1 antibody	9 (17.3)
Anti–PD-1/CTLA-4 antibody	3 (5.8)
Treatment duration, wk	
0-4	27 (51.9)
5-9	8 (15.4)
≥10	17 (32.7)
Documents per patient, No.^a^	
1-50	13 (25.0)
51-100	17 (32.7)
101-200	11 (21.1)
≥201	11 (21.1)

Abbreviations: CTLA-4, cytotoxic T-lymphocyte antigen 4; PD-1, programmed cell death 1; PD-L1, programmed cell death ligand 1.

^a^ Documentation included oncology clinic notes, telephone encounters, images, and hospitalization records. Laboratory results were not included in the documentation count.

**Figure 1.**
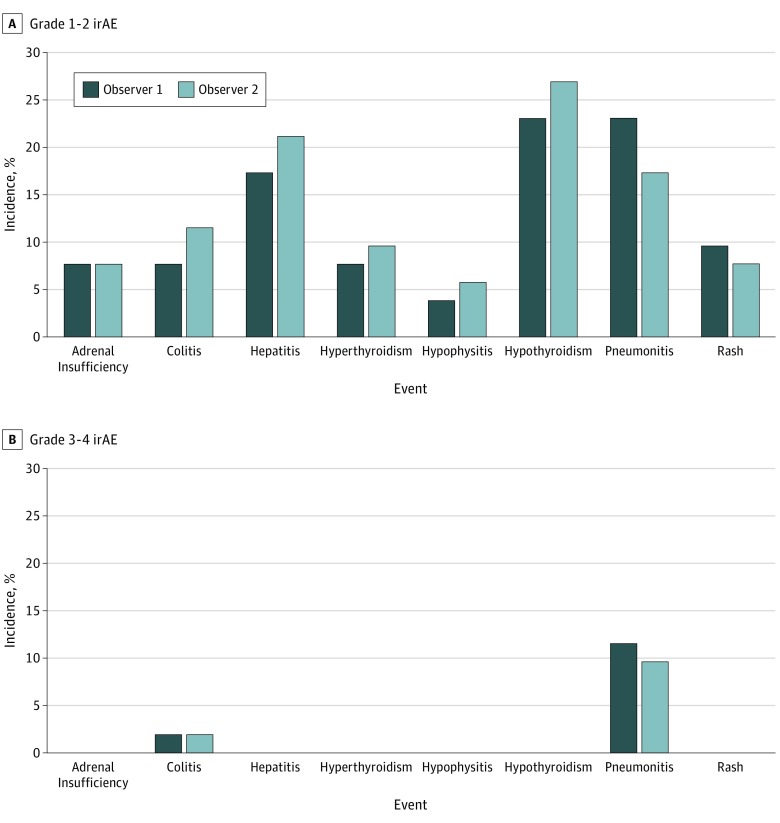
Incidence and Grading of Immune-Related Adverse Events (irAE) by Each Observer

**Figure 2.**
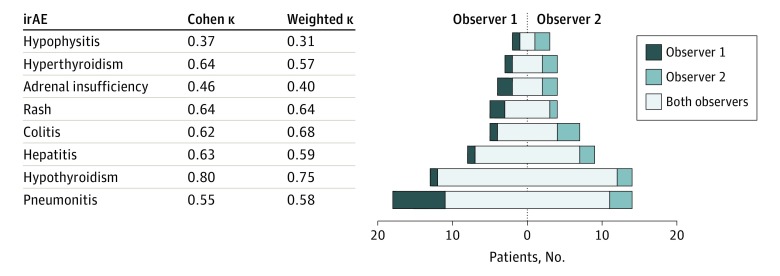
Immune-Related Adverse Events (irAE) Identified by Each Observer The Cohen κ reflects agreement on irAE occurrence. Weighted κ reflects agreement on irAE grade.

Frequency of irAE ranged from 4% to 35% for observer 1 and from 6% to 27% for observer 2, with pneumonitis the most common and hypophysitis the least common for both observers (Figure 1). Combining assessments of both reviewers, irAE incidence ranged from 8% (hypophysitis) to 40% (pneumonitis). Considering only those cases for which both observers identified a specific irAE, irAE incidence ranged from 2% (hypophysitis) to 21% (pneumonitis) (eTable in the Supplement). In general, neither observer demonstrated consistent underreporting or overreporting of irAE compared with the other. Figure 2 shows the agreement on irAE occurrence (Cohen κ) and irAE grading (weighted κ). Overall, there was limited or poor agreement on irAE occurrence (Cohen κ, 0.37-0.64), with the exception of hypothyroidism (κ = 0.8). Agreement on irAE grading was similarly limited (weighted κ, 0.31-0.75). Overall, the highest grade for an irAE by either observer was grade 1 in 64%, grade 2 in 17%, and grade 3 to 4 in 15% of cases. Agreement was greater for grade 3 to 4 (80%) than for grade 1 (61%) or grade 2 (59%) toxic effects. Observer assessments of irAE occurrence and grading for individual patients are shown in the eFigure in the Supplement. We also observed notable differences in the recorded date of irAE onset (range, 5-188 days) (eTable in the Supplement). As a control for data availability and access, we observed a high degree of agreement between observers for the exact start date (98%) and end date (96%) of immunotherapy administration.

Case characteristics associated with discordant assessment of irAE occurrence are shown in Table 2. As in the univariable model, in multivariable analysis, therapy duration (adjusted odds ratio, 4.80; 95% CI, 1.34-17.17; *P* = .02) and Charlson Comorbidity Index (adjusted odds ratio, 4.09; 95% CI, 1.10-15.18; *P* = .03) were significantly associated with discordant irAE assessment. There was no association with number of documents reviewed or history of autoimmune disease.

**Table 2.** Factors Associated With Discordant Immune-Related Adverse Event Assessment

Case Characteristic	No. of Cases	Univariable Analysis		Multivariable Analysis	
		OR (95% CI)	*P* Value	OR (95% CI)	*P* Value
Therapy duration, d					
≤50	26	1 [Reference]	.01	1 [Reference]	.02
>50	26	4.34 (1.34-14.03)		4.80 (1.34-17.17)	
No. of documents					
≤100	30	1 [Reference]	.88	1 [Reference]	.88
>100	22	1.10 (0.36-3.30)		0.91 (0.26-3.21)	
Charlson Comorbidity Index					
≤9	33	1 [Reference]	.03	1 [Reference]	.03
>9	19	3.58 (1.10-11.63)		4.09 (1.10-15.18)	
Autoimmune history					
No	43	1 [Reference]	.51	1 [Reference]	.35
Yes	9	0.60 (0.13-2.71)		0.39 (0.06-2.80)	

Abbreviation: OR, odds ratio.

## Discussion

Years into the remarkable era of cancer immunotherapy, irAE continue to plague patients and puzzle clinicians. To understand the challenges of diagnosing and characterizing these autoimmune toxic effects, this study assessed observer agreement on irAE occurrence, type, grade, and timing. As individual clinicians are chiefly responsible for the identification, reporting, and management of treatment toxic effects in everyday practice and in clinical trials, our assessment of individual observer reporting provides highly relevant insights into irAE detection and assessment. We found substantial disagreement (κ < 0.7, which indicates an explained variance of less than 50%) between 2 experienced medical oncologists for all irAE, with the exception of hypothyroidism (κ = 0.8).^14^ As a clinical entity among irAE, therapy-related hypothyroidism is uniquely characterized by well-defined laboratory correlates and no other likely etiology during treatment. Other irAE either lack laboratory correlates (eg, pneumonitis) or may have nonimmune causes during therapy (hepatitis), complicating their assessment.

The incidence of specific irAE reported by each observer was comparable to other real-world settings, although some were consistently higher than reported rates in clinical trials.^7,8,9^ These differences could reflect the detailed, algorithm-driven, multidisciplinary review of cases we performed. Alternatively, our real-world patient cohort may be less fit than highly selected clinical trial populations.^9^ However, our cohort demographic characteristics are comparable to those reported in multiple phase 3 lung cancer trials investigating immunotherapy.^15,16^

Case characteristics associated with discordant irAE assessment—longer treatment periods and greater comorbidity burden—all reflect clinical complexity. Interobserver agreement was greatest for higher-grade irAE, which may indicate both relative ease of detection and lack of alternative explanations. Nevertheless, irAE considered low grade—in contrast to most grade 1 to 2 chemotherapy toxic effects such as myelosuppression—may represent truly clinically significant events. For instance, grade 2 pneumonitis includes events that limit instrumental activities of daily living. Grade 2 colitis includes gastrointestinal bleeding.

Importantly, a number of studies have suggested that patients who develop irAE may be more likely to exhibit objective responses and prolonged overall or progression-free survival.^17,18,19,20^ Among patients who develop irAE, having multiple or higher-grade irAE may further stratify patients who benefit the most from immunotherapy.^21^ Notably, the precise method of irAE abstraction was not thoroughly detailed in these studies, and rates of irAE varied considerably. For instance, among these studies, pneumonitis incidence ranged from 2% to 18% in cohorts of Japanese patients with lung cancer who were treated with nivolumab.^17,18,19,20^ Given the potential role of irAE as prognostic or predictive markers, our work highlights an emerging need for developing transparent and standardized approaches to irAE identification to guide clinical care beyond the management of toxic effects.

The lack of an association between discordant irAE assessment and case documentation volume suggests that poor interrater reliability is not chiefly a function of observer negligence, in which case discordance would be expected to increase with observer effort. Further confirmation of consistent data collection is apparent through the high degree (>95%) of interrater agreement in the assessment of exact immunotherapy administration dates. Instead, the association of discordant irAE assessment with therapy duration could reflect the increasing risk of irAE as immunotherapy exposure increases. Discordant irAE assessment was also associated with comorbidity burden, which could reflect confounding by laboratory, radiology, and clinical abnormalities of non-irAE etiology. As patients with multiple comorbidities are common in oncology practices, future efforts to improve irAE detection and characterization of irAE in these individuals are particularly critical. Potential strategies include embedding more than 1 observer in the assessment or review of irAE and using automated tools and collateral data to reduce physician effort.^22,23^

Our findings may also point to the importance of multidisciplinary evaluation and management. Might pulmonologists have more frequently agreed on pneumonitis cases? Would endocrinologists be more consistent in their assessment of adrenal or pituitary dysfunction? Clearly, irAE require broader clinical input than do toxic effects of conventional chemotherapy or molecularly targeted therapy. Having ready access to a multidisciplinary team that includes rheumatologists, pulmonologists, endocrinologists, gastroenterologists, and other specialists is critical for any medical oncologist, primary care practitioner, and emergency department clinician who may be the first medical professional to encounter a patient experiencing acute irAE.

### Limitations

Limitations of this analysis include the single-center setting, the preponderance of lung cancer cases, and the limited number of cases treated with anti–CTLA-4 therapies. This study was also restricted to examining 8 irAE, which we selected based on their incidence and established association with immunotherapy. Reporting characteristics for other irAE (such as nephritis) may differ, but we suspect would have comparable rates of interrater reliability based on the complexity of diagnosis, effort needed to capture events, and incidence.^22^ Additionally, in real-world clinical practice, irAE are diagnosed and treated in real time, rather than retrospectively. However, retrospective analysis has traditionally been applied to the adjudication of events within and between clinical trials. Furthermore, if anything, one might expect an even greater degree of discordance with real-time assessment, as clinicians managing acutely ill patients may not have the time, algorithm, or degree of documentation available to the clinical reviewers in this study. In addition, we recognize that the clinical reviewers in this study have not served as study chairs or principal investigators on national or international immunotherapy clinical trials. However, their years of experience in the administration of immune checkpoint inhibitors to dozens of patients with diverse cancer types both on and off protocol render them highly representative of most medical oncologists using these agents.

## Conclusions

In conclusion, this study highlights the challenges of recognizing and characterizing irAE. While most toxic effects of conventional chemotherapy and molecularly targeted therapies are readily diagnosed through medical history, physical examination, and laboratory data, irAE appear far more heterogeneous and unpredictable. Variability in the presentation and timing of irAE makes it difficult for clinicians to attribute properly unpredictable irAE signs or symptoms to drug toxicity rather than other causes. Current laboratory and imaging correlates do not discriminate between immune- and nonimmune-related etiologies, and the lack of specific immune biomarkers contributes to the challenges of capturing irAE by clinicians. Interobserver differences in irAE incidence suggest limitations of cross-study toxic effect comparisons. However, even when rates of irAE are similar, precisely which patients experience these events remains discordant, providing evidence that concerns about the reliability of irAE diagnosis and characterization extend beyond clinical research into real-world, day-to-day clinical practice. Indeed, the inappropriate withholding or continuation of immune checkpoint inhibitor therapy—not to mention the risks of inappropriate exposure to high-dose, prolonged glucocorticoids—carries considerable clinical implications. While discrepancies between nonphysician- and physician-reported AE are known to be pervasive,^22,24^ we have demonstrated substantial interphysician discordance for the assessment of irAE despite use of a consistent and methodical approach to case evaluation. Whether advances in information technology, such as automated medical record abstraction,^25^ will improve irAE identification and reporting is not yet clear. The present work advocates for greater awareness and multidisciplinary involvement in the diagnosis and management of irAE, the development of well-defined diagnostic and grading criteria, and—eventually—the development of biomarkers for the prediction, diagnosis, and tracking of irAE.

## Notes

Supplement. **eFigure.** Type and Grade of Individual Immune-Related Adverse Events (irAE) Identified by Each Observer **eTable.** Incidence and Timing of Immune-Related Adverse Events (irAE)
