# Supplementary material for: Variation in the Assessment of Immune-Related Adverse Event Occurrence, Grade, and Timing in Patients Receiving Immune Checkpoint Inhibitors
Source: JAMA Netw Open. 2019 Sep 18;2(9):e1911519. doi: 10.1001/jamanetworkopen.2019.11519 (PMC6751757; doi:10.1001/jamanetworkopen.2019.11519)
Supplement: Supplement. — eFigure. Type and Grade of Individual Immune-Related Adverse Events (irAE) Identified by Each Observer eTable. Incidence and Timing of Immune-Related Adverse Events (irAE) [file jamanetwopen-2-e1911519-s001.pdf]

Supplementary Online Content

Hsiehchen D, Watters MK, Lu R, Xie Y, Gerber DE. Variation in the assessment of immune-related adverse event occurrence, grade, and timing in patients receiving immune checkpoint inhibitors. *JAMA Netw Open*. 2019;2(9):e1911519. doi:10.1001/jamanetworkopen.2019.11519

eFigure. Type and Grade of Individual Immune-Related Adverse Events (irAE) Identified by Each Observer

eTable. Incidence and Timing of Immune-Related Adverse Events (irAE)

This supplementary material has been provided by the authors to give readers additional information about their work.

© 2019 Hsiehchen D et al. *JAMA Network Open.*

eFigure. Type and grade of individual immune-related adverse events (irAE) identified by each observer. The size of each symbol reflects the irAE grade.

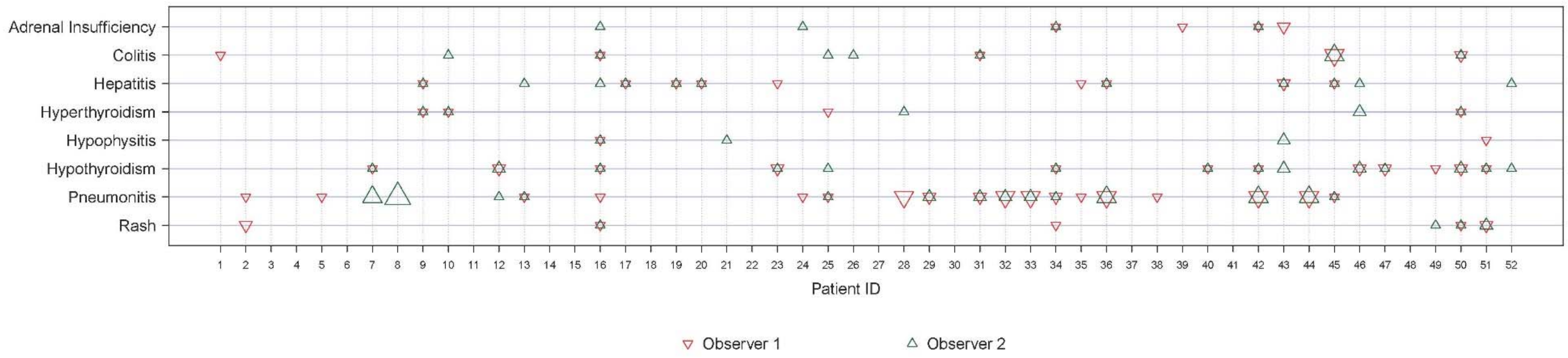

© 2019 Hsiehchen D et al. *JAMA Network Open.*

eTable. Incidence and timing of immune-related adverse events (irAE).

	Combined incidence† (%)	Overlapping incidence‡ (%)	Mean time to onset* (Observer 1) (days)	Mean time to onset* (Observer 2) (days)	Mean difference in time to onset* (days)
Adrenal Insufficiency	11.5	5.8	113	276	187
Colitis	15.4	7.7	157	254	5.3
Hepatitis	25	13.4	113	98	25
Hyperthyroidism	9.6	5.8	225	119	110
Hypophysitis	7.7	1.9	99	95	0**
Hypothyroidism	28.8	23	116	113	45
Pneumonitis	40.4	21	123	251	76
Rash	11.5	5.8	248	295	61

†Includes cases identified by either or both observers.

‡Includes cases identified by both observers.

*Calculated from the date of first infusion of immune checkpoint inhibitor therapy.

******For the single overlapping case of hypophysitis identified by both observers, the time to onset was identical.

© 2019 Hsiehchen D et al. *JAMA Network Open.*
